# Perceptions about Eclampsia, Birth Preparedness, and Complications Readiness among Antenatal Clients Attending a Specialist Hospital in Kano, Nigeria

**DOI:** 10.1155/2015/431368

**Published:** 2015-07-14

**Authors:** Umar Muhammad Lawan, Idris Usman Takai, Hamza Ishaq

**Affiliations:** ^1^Department of Community Medicine, Bayero University and Aminu Kano Teaching Hospital, Kano 700001, Nigeria; ^2^Department of Obstetrics and Gynaecology, Bayero University and Aminu Kano Teaching Hospital, Kano 700001, Nigeria; ^3^College of Health Sciences, Bayero University, Kano 700001, Nigeria

## Abstract

*Background.* Eclampsia is a reliable indicator of poor birth preparedness and complications readiness. We determined perceptions about eclampsia, birth preparedness, and complications readiness among antenatal clients in Kano, Nigeria. *Materials and Method.* A cross-sectional design was used to study 250 randomly selected antenatal clients. Data was analyzed using SPSS 16.0. *Result.* The mean age of the respondents was 26.1 ± 6.4 years. The majority perceived that eclampsia is preventable through good ANC (76.4%) and hospital delivery (70.8%). Overall, 66.8% had good perception about eclampsia. Having at least secondary school education and multigravidity were associated with good perception about eclampsia on multivariate analysis. About a third (39.6%) of the mothers was less prepared. On binary logistic regression, good perception about eclampsia and multigravidity were associated with being very prepared for birth. Up to 37.6% were not ready for complications. Half (50.4%) knew at least three danger signs of pregnancy, and 30.0% donated blood or identified suitable blood donor. On multivariate analysis, having at least secondary school education, being very prepared for birth, and multigravidity emerged as the only predictors of the respondents' readiness for complications. *Conclusion and Recommendations.* Health workers should emphasize the practicability of birth preparedness and complications readiness during ANC and in the communities, routinely review plans, and support clients meet-up challenging areas. The importance of girl-child education to at least secondary school should be buttressed.

## 1. Introduction

Eclampsia is a disturbance of the central nervous system in obstetric patient(s) characterised by seizure activity or coma with or without a prior history of hypertension in pregnancy, proteinuria, and/or oedema (preeclampsia) [1–3]. Eclampsia may occur during pregnancy, child birth, or as long as 3 weeks after birth.

Both preeclampsia and eclampsia account for significant maternal and fetal morbidity and mortality. Eclampsia alone accounts for approximately 50,000 maternal deaths worldwide annually [4]. Its reported incidence ranges from 1 in 100 to 1 in 3448 pregnancies globally [5]. Eclampsia is one of the leading causes of maternal mortality in Nigeria where as many as 545 maternal deaths per 100,000 live births occur annually [1, 2, 6]. The social burden of eclampsia is more marked in less developed nations especially in Africa where precipitating cultures and traditions like early marriage, short birth intervals, hot bath, and use of high osmolar potash gruel/drinks for immediate postpartum mothers prevail; maternal mortality is high and there is poor access to quality antenatal care and essential obstetric care services. Apart from the physical illness and psychological stress from the condition, victims and their families are often stigmatized and discriminated due to the prevailing perceptions that the condition runs in families, it is caused by evil spirits, it occurs as a punishment from God for bad deeds, or because it is perceivably associated with witches and wizards [7–9].

Scientifically, eclampsia is an indicator of poor birth preparedness and complication readiness [10]. Birth preparedness and complication readiness are a strategy designed to promote the timely use of skilled maternal and neonatal care especially during childbirth and on the premise that preparing for childbirth reduces delay in obtaining this care [6]. Birth and emergency preparedness plans should be discussed on first ANC visit, revised by subsequent visits, and finalized by 32nd week of gestation [11]. Birth plan/emergency preparedness plan encompasses the mothers' knowledge of danger signs; the desired place of birth; the birth attendant; and the location of the closest appropriate care facility. Other components of the plan include setting aside funds for birth-related and emergency expenses; a birth companion; support in looking after home and children while being away; transport to a health facility for the birth; transport in case of obstetric complication, and identification of compatible blood donors in case of emergency [6, 7, 12].

Empirical evidence suggests wrong perceptions about eclampsia in Nigeria and other parts of Africa [7–9], and mothers are often less prepared for deliveries or unexpected eventualities associated with pregnancy and child birth [1, 13–15]. This study was therefore to determine perceptions about eclampsia and level of birth preparedness and readiness to complications among antenatal clients attending a specialist hospital in Kano, Nigeria. We used the health belief model to conceptualize how the antenatal clients' perceptions of eclampsia as a proxy for obstetric emergencies influenced their planning for birth and emergencies. Findings from this study would be useful for clinicians, MNCH program managers, and researchers for designing ANC specific and community directed interventions aimed at reducing maternal mortality and morbidity in Nigeria and beyond.

## 2. Materials and Method

### 2.1. Setting

The study was conducted in Murtala Muhammad Specialist Hospital (MMSH) located within the ancient Kano city. The hospital serves the inhabitants of Kano state, surrounding states and people from neighboring Nigeria. MMSH has a bed capacity of about 250 and a monthly patients' turnover of about 18,000. About 1400 clients attend the facility for antenatal care each month.

### 2.2. Study Design, Subjects, and Sample Size

We used a cross-sectional design to study a random sample of 250 antenatal clients attending the ANC clinic in MMSH. The sample size was determined using an appropriate statistical formula for estimating minimum sample size for descriptive studies [16] and 13.4% proportion of women with good perception of eclampsia from a study conducted among major ethnic groups in Borno State, Nigeria [7]. The calculated sample size of 178 was inflated to 250 to compensate for incomplete responses and other contingencies.

### 2.3. Sampling

A systematic sampling technique was used for selection of the subjects based on the monthly clinic attendance of 1400 and using the ANC register. With a sampling interval of 6 (i.e., 1400/250), 1 in every 6 ANC clients was selected until the required sample size was met. The starting point for selection was determined by picking a random number from the clinic register between 1 and 6. Subsequent respondents were identified by adding the sampling interval to the preceding respondents' serial number on the ANC register until the required sample size was met.

### 2.4. Instrument and Method of Data Collection

Pretested semistructured interviewer-administered questionnaires were used for data collection. The questionnaire consisted of three sections that elicited the sociodemographic characteristics of the respondents, perception of the clients on causes/risk factors, prevention and management of eclampsia, and birth preparedness and complications readiness of the clients. The questionnaires were administered by four trained interviewers after obtaining informed consent from the mothers. Literate respondents indicated acceptance by signing the consent form, while nonliterate participants affixed their thumbprint. Permission and ethical clearance for the study were also sought and obtained from MMSH and Ethical Committee of Aminu Kano Teaching Hospital, respectively. Data was collected in August/September 2014.

### 2.5. Data Analysis

Data were analyzed using SPSS version 16.0 computer statistical software. Absolute numbers and simple percentages were used to summarize categorical variables, whereas quantitative variables were summarized using means and standard deviation. The clients' perceptions about eclampsia and birth preparedness and complications readiness were scored and graded using a scoring system adapted from a past study where each correct answer was given one point and wrong response was allocated no point [17]. Accrued points were subsequently graded in percentages. Out of the total of 17 points for perception, respondents that scored 10 to 17 points were adjudged to have “good perception,” whereas those that scored 0 to 9 points had “poor perception,” In the same vein out of a total of 8 points for preparedness, clients that scored 5 to 8 points were considered to be “very prepared” while those that scored 0 to 4 points were “less prepared.” For complications readiness, clients that scored 4 to 7 points out of the total of 7 points were considered to be “ready” for complications whereas those that scored 0 to 3 points were “not ready.”

The chi-square test and Fisher's exact probability test were used for bivariate analysis involving categorical variables, and binary logistic regression was used to determine factors that predict good perception of eclampsia, being very prepared for birth, and being ready for complications. A *p* value ≤ 0.05 was considered statistically significant.

## 3. Results

### 3.1. Sociodemographic Characteristics

The respondents' ages ranged from 15 to 40 years with a mean and standard deviation 26.1 ± 6.4 years. The majority of them were in the age bracket 20 to 24 years (29.6%), from Hausa and Fulani ethnic groups (219) (87.6%), married (242) (98.6%), unemployed women (169) (67.6%), and with no secondary school level of education (175) (72.0%) as summarized in Table 1.

### 3.2. The ANC Clients' Perceptions about Eclampsia

The parameters used for assessing the ANC clients' perceptions about eclampsia are as summarized in Table 2. All the respondents had heard about eclampsia or seen some one with the condition. Up to three-quarters (75.6%) believed that it is possible to identify causes or risk factors of developing eclampsia in pregnancy. A significant proportion of them correctly perceived that preexisting hypertension and poor prenatal care are risk factors of the disease. Interestingly, however, some of the ANC clients wrongly perceived that eclampsia occur as a destiny from God (51.2%) or that it is inflicted upon the clients by evil spirits (18.8%). Commendably the majority of the clients perceived that eclampsia is preventable and that good antenatal care (76.4%) and hospital delivery (70.8%) are recognized preventive measures against the condition.

More than three-quarters of the clients (78.0%) perceived that eclampsia can be managed effectively. However, 29.6% and 6.0% claimed that it is best managed through spiritual means/seeking for divine intervention or through a combination of hospital care and divine intervention/spiritual, respectively.

Overall, when the respondents were cumulatively assessed on their perceptions about eclampsia, it was observed that 167 (66.8%) had good perceptions about the condition (Table 2). This study observed that age of the clients more than 24 years, Hausa/Fulani ethnicity, having at least secondary school level of education, and multigravidity were significantly associated with the clients' good perception about eclampsia. On binary logistic regression in a model consisting of these variables, having at least secondary school level of education and multigravidity emerged as the only client related factors associated with good perception about eclampsia (Table 5).

### 3.3. Birth Preparedness and Complications Readiness of the ANC Clients

We assessed the respondents' birth preparedness using nine (9) indices outlined in Table 3. Most of the respondents could identify correctly the signs of normal labour (92.4%) and had agreed with their spouses on choice of place of delivery (79.2%), how to get to the desired place of delivery in case of labour (73.2%), making arrangement for companion to hospital in case of labour (77.6%), and also saving some money for the purpose of transportation to the desired place of delivery, labour, and the accompanying expenses (84.4%). However, only half (56.4%) of the clients went ahead to identify the means of transportation to the hospital when labour starts or made a contingency arrangement on how to get to the place of delivery when the identified means of transportation fails (47.2%). Additionally only about a third (38.4%) of the clients had standing permission from their spouses on getting to the place of delivery when labour starts in their absence. Overall, when the clients were scored based on the preparedness indices, it was observed that about a third of them (79) (39.6%) were less prepared (Table 3). Being very prepared for birth was significantly associated with having good perception about eclampsia and multigravidity. On binary logistic regression, using a model consisting of the respondents' perception about eclampsia, multigravidity, and other important maternal factors including age, ethnicity, and educational status, good perception about eclampsia and multigravidity still emerged as the significant factors that were associated with being very prepared for birth (Table 5).

The ANC clients' readiness for complications was assessed using seven (7) indices outlined in Table 4. Except for the overlapping activities of preparation for normal delivery like saving money for delivery or arranging for companionship to hospital, the clients did not perform most of the indices used to assess their readiness for unexpected complications. Only about half of them (50.4%) knew at least three (3) danger signs of pregnancy, and less than one-third (30.0%) donated blood or identified suitable blood donor in case of emergency. Interestingly, most of the clients examined (94.4%) had made contingency arrangement with a health worker in case of unexpected delivery at home. This study also observed that more than one-third of the clients (37.6%) were not ready for unexpected events during pregnancy or at point of labour (Table 4). On bivariate analysis, clients' readiness for complications was found to be significantly associated with having good perception about eclampsia, being very prepared for birth, older age (age > 24 years), multigravidity, having at least secondary school level of education, and being from Hausa/Fulani ethnicity. When these variables were ran in multivariate analysis, having at least secondary school level of education, being very prepared for birth, and multigravidity emerged as the only predictors of the respondents' readiness for complications (Table 5).

## 4. Discussion

Antenatal care (ANC) is an important component of preventive care services and those health care providers offering this service can effectively lower and/or eliminate the risk of developing complications associated with pregnancy and delivery through antenatal health care talk (counseling) and possibly by the use of some forms of interventions.

### 4.1. Perceptions about Eclampsia

The major objective of antenatal care is to ensure optimal health outcomes for the mother and her baby. In our study, up to 76.4% of our respondents perceived that the risk of developing eclampsia can be prevented by good ANC as well as hospital delivery (70.8%). Eclampsia has been documented as one of the leading causes of maternal mortality in most Nigerian and other sources of the literature [1, 2, 4–9, 18, 19], and lack of awareness and/or utilization of maternal health care service are identified as leading risk factors for its occurrence [10, 18, 19]. According to Omotara [20], utilization of health care services in general is affected by awareness of the health care seekers. This is because women and their families lack understanding of the danger signs or gravity of the condition or because they do not know where to go to seek help in times of need. This invariably affects their abilities to make informed decision about seeking healthcare and as a result constrains them from exercising their reproductive rights. The average maternal mortality ratio for Kano state in northwestern region of Nigeria is 1600/100,000 live births [19]. This is almost 3 times higher than the national average of 545/100,000 live births [1, 2, 6, 18] and is one of the worst ratios in the world. The 70% wrong perception of eclampsia in our study further buttresses eclampsia as the leading cause of maternal mortality in our locality. This may not be unconnected with the level of higher education of the mothers as only 1.6% of them had postsecondary school education. This finding is particularly important because it reflects the need for improvements in the quality of maternal health care services received in our community. Furthermore, reports indicate that more than one-third of the population of Nigeria live in extreme poverty—defined by the World Bank as earnings of under $1 per day, while 9 out of 10 Nigerians live on less than $2 per day [21]. The zones in the northern part of the country where this study was carried out present the highest vulnerability, with highest proportion of the households experiencing chronic poverty [21]. This report is in keeping with the finding of this study in that majority of our respondents (67.6%) were not gainfully employed, and this may perhaps explain why more than one-third of them will seek spiritual and/or divine intervention in the management of eclampsia.

In our study, overall level of good perceptions regarding eclampsia was very high (66.8%) when compared with only 13.4% reported from a study conducted in Borno State, northeastern part of the country [7]. This disparity may perhaps be associated with higher exposure of our respondents to maternal and child health issues, either from their educational attainment or from experiences surrounding their high parity. Our findings indicated that those respondents having at least secondary school level of education and multigravidity were more likely to have good perception about eclampsia than their counterparts. They may have perhaps accrued more experience from repeated child bearing and ANC exposures. The high level of good perception of eclampsia recorded in this study is quite commendable because it influenced their health seeking behavior [20]. This is corroborated by the fact that more than three-quarters of the clients studied perceived correctly that eclampsia can be prevented and managed effectively in the hospital.

### 4.2. Birth Preparedness and Complications Readiness

Birth preparedness is a fundamental component of antenatal care whose aim is to reduce unnecessary delays to seek emergency obstetric care in order to improve maternal and fetal outcomes [15]. Our respondents' performance on the indices used to assess birth preparedness was high, remarkable, and similar to a multicentre study conducted in Nigeria, where it was reported that awareness of the concept of birth preparedness was high [22]. Most of our respondents (92.4%) correctly identified the signs of labour and had agreed with their spouses on choice of place of delivery (79.2%). This is particularly commendable because being aware of when labour starts is a positive step towards activation of the birth preparedness and complication readiness plan. Past studies from parts of Africa and Asia reported lower levels of this stage of preparedness [14, 15, 23]. It is thus critical that all ANC attendees are well knowledgeable about signs of labour in order to gain easy access to skilled attendance at labour and delivery and to reduce and/or eliminate associated complications. Our study also observed that majority of our respondents had saved money for the purpose of transportation to their desired place of delivery. More than half had identified means of transportation to the hospital, and three-quarters had made arrangement for companionship. Similar levels of preparations were reported in previous studies [14, 15, 24]. Sadly, only about a third of our respondents had standing permission from their spouses on getting to the place of delivery when labour starts in their absence. This is not surprising because the majority of the clients studied (87.6%) were from the Hausa/Fulani ethnic group and predominantly Muslims among whom the practice of “purdah” which isolates the woman and encourages subjugation and overdependence on the man is common [18, 25]. In some cultures a woman cannot go to the hospital without the husband's prior permission; thus, even if she is pregnant or in labour and a complication arises, she may likely die at home if her husband is away [18, 25]. Some religions encourage fatalism by attributing every misfortune to the Almighty, thereby unnecessarily resigning themselves to fate rather than take positive steps to help themselves [18, 25].

Overall, up to 39.6% of the clients we examined were less prepared. This proportion is still lower than the 65% less prepared reported from a community-based study from Uganda [26] but slightly higher than the 28.3% reported from southern part of Nigeria [27]. This difference may be a result of sociocultural differences of the populations and perhaps the scale on which the key indices of birth preparedness were assessed.

We also observed that respondents' good perception about eclampsia and multigravidity was strong predictors of birth preparedness. This was similarly reported from Delhi [23] and other parts of Nigeria [27].

Except for the overlapping activities of preparations for normal delivery like saving money for delivery or arranging for companionship to hospital, our respondents did not perform well on most of the indices used to assess their readiness for unexpected complications. While most study participants (94.4%) viewed the occurrence of obstetric complications as a serious matter and noted the importance of saving funds in case of emergencies and had made contingency arrangement with a health worker in case of unexpected delivery at home, more than a third of them (37.6%) were not ready for unexpected events during pregnancy or at point of delivery. This clearly shows that despite high awareness of the need to have funds available, some of our respondents were not able to amass any savings. This is especially true in our locality with high level of poverty [21], where in most cases, poor patients and their families face serious challenges with mobilizing money in cases of emergencies.

Knowledge of the danger signs of obstetric complications is an essential step in recognition of complications and enables one to take appropriate action to access emergency care [10]. Only about half (50.4%) of our respondents knew at least three danger signs of pregnancy; this is in spite of the fact that up to 92.4% of them correctly identified the signs of normal labour. Our finding is commendable and higher than the 6.7% that knew three or more danger signs reported from a previous study from Asia [15]. Although less than one-third (30%) of our respondents donated blood or identified suitable blood donor in case of emergency, the finding is comparable to the 28.7% and 27.8% reported from parts of Asia [15, 23].

We also found that having at least secondary level of education (*p* = 0.009), being very prepared for birth (*p* = 0.0001), and multigravidity (*p* = 0.0001) emerged as the only predictors of the respondents' readiness for complications on multivariate analysis. This finding agrees with that of Hiluf and Fantahun from Ethiopia, where preparation for birth and its complication was found higher among literate mothers, women with parity range of 2 to 4, women with history of stillbirth, and those who were advised about birth preparedness during their antenatal care follow-up [28]. Similarly, a previous study from southeastern Nigeria reported that educational status and high parity of the respondents were the best predictors of awareness of the concept of birth preparedness and complication readiness [24]. Birth preparedness and complication readiness as components of focus antenatal care are an important concept that will significantly reverse the ugly trend of maternal and perinatal morbidity and mortality experienced in our community if strictly followed. Birth preparedness and complication readiness involve not only the pregnant woman, but also her family, community, and available health care providers and hence the need for collaborative efforts of this group of people for a robust, effective, and efficient health care delivery.

Based on the pertinent findings of our study, we recommend that health care workers should strengthen birth preparedness and complications readiness messages by emphasizing their practicability and reinforce this through repeated health education in hospitals during ANC and in the communities. They should routinely review progress made on both components of the plan on each ANC visits and support clients to meet challenging areas. The importance of girl-child education to at least secondary school level should be buttressed during these campaigns.

## Figures and Tables

**Table 1:** Respondents' social and demographic characteristics.

Characteristic	Frequency (%) (*n* = 250)
Age (years)	
15–19	38 (15.2)
20–24	74 (29.6)
25–29	57 (22.8)
30–34	46 (18.4)
39-40	35 (14.0)
Education	
No education	20 (8.0)
Quranic only	75 (30.0)
Primary	80 (32.0)
Secondary	71 (28.0)
Postsecondary	4 (1.6)
Parity	
Primigravida	58 (23.2)
Multigravida	117 (46.8)
Grand multigravida	75 (30.0)
Ethnicity	
Hausa	179 (71.6)
Fulani	40 (16.0)
Yoruba	9 (3.6)
Igbo	6 (2.4)
Others	16 (6.4)
Marital status	
Married	242 (96.8)
Widowed	8 (3.2)
Occupations	
Unemployed	169 (67.6)
Petty trading	77 (30.8)
Civil service	2 (0.8)
Employed in private sector	2 (0.8)

**Table 2:** Assessment of the ANC clients' perception about eclampsia.

Parameters/indices	Frequency (%) (*n* = 250)
Believe it is possible to identify causes and risks of developing eclampsia^††^	189 (75.6)
Perceptions about the causes/risk factors of eclampsia^†^	
Preexisting hypertension	155 (62.0)
Poor prenatal care	111 (44.4)
Runs in families	35 (14.0)
Poor nutrition	46 (18.4)
Destiny	128 (51.2)
Evil spirits	47 (18.8)
Believe eclampsia is preventable^††^	210 (84.0)
Perceptions about ways of preventing eclampsia^†^	
Good antenatal care	191 (76.4)
Hospital delivery	177 (70.8)
Avoiding early marriage	28 (11.2)
Avoiding delivery at ≥35 years	9 (3.6)
Believe that eclampsia can be effectively managed^††^	195 (78.0)
Perceptions about the best source of treatment for eclampsia^†^	
Hospital	161 (64.4)
Spiritual/divine intervention	74 (29.6)
Hospital and divine/spiritual intervention	15 (6.0)

Grading of the ANC clients' perception about eclampsia	
Good (10–17)	167 (66.8)
Poor (0–9)	83 (33.2)

† refers to multiple responses: each of the responses given is out of the 250 sample.

†† refers to nonmultiple responses.

**Table 3:** Assesment of the ANC clients' Birth preparedness.

Parameters/indices	Frequency (%) (*n* = 250)
Can identify signs of normal labour	231 (92.4)
Agreed with spouse on place of delivery	198 (79.2)
Decided with spouse on how to get to place of delivery	183 (73.2)
Saved money for transport and delivery	211 (84.4)
Identified means of transport to desired place of delivery	141 (56.4)
Had made contingency plan on how to get to delivery place if identified means fails	118 (47.2)
Had obtained standing permission from spouse to get to place of delivery	96 (38.4)
Had made arrangement for companion(s) to place of delivery	194 (77.6)

Respondents' grading of Birth preparedness	
Very prepared (5–8)	171 (68.4)
Less prepared (0–4)	79 (39.6)

**Table 4:** Assessment of the ANC clients' complications readiness.

Parameters/indices	Frequency (%) (*n* = 250)
Knew at least 3 danger signs of pregnancy	126 (50.4)
Donated blood/identified suitable blood donor(s)	75 (30.0)
Saved money for transportation and delivery or in case of complication(s)	211 (84.4)
Obtained standing permission from spouse to go to hospital in case of labour or any complication(s)	96 (38.4)
Identified means of transportation to get to the hospital in case of labour or any complication(s)	141 (56.4)
Identified suitable person(s) to accompany them to hospital in case of labour or complication(s)	194 (77.6)
Had made contingency arrangement with a health worker in case of unexpected delivery at home	236 (94.4)

Grading of the ANC clients readiness to complications	
Ready (4–7)	156 (62.4)
Not ready (0–3)	94 (37.6)

**Table 5:** Factors associated with the ANC clients' perceptions about eclampsia, birth preparedness, and complications readiness.

Characteristic	Bi-variate			Binary logistic regression	
(A) Good perception about eclampsia	(*n* = 167) Frequency (%)	Total	Statistical test (*p* value)	*Z* test (*p* value)	OR (95% C.I.)

Age >24 years	101 (60.5)	138	*χ* ^2^ = 5.67 (0.017)	1.44 (0.149)	
Hausa/Fulani ethnicity	138 (82.6)	219	Fisher's (0.0002)	1.62 (0.106)	
Having at least secondary education	59 (35.3)	75	*χ* ^2^ = 6.80 (0.0009)	2.57 (0.010)	3.06 (1.30, 7.16)
Multigravidity	136 (81.4)	192	*χ* ^2^ = 6.07 (0.014)	2.20 (0.028)	2.67 (1.11, 6.37)

(B) Birth preparedness (very prepared)	(*n* = 171) Frequency (%)	Total	Statistical test (*p* value)	*Z* test (*p* value)	OR (95% C.I.)

Having good perception about eclampsia	129 (75.4)	167	*χ* ^2^ = 18.21 (0.0001)	3.17 (0.002)	2.86 (1.49, 5.49)
Age >24 years	101 (59.1)	138	*χ* ^2^ = 3.27 (0.071)		
Hausa/Fulani ethnicity	146 (85.4)	219	Fisher's test (0.080)		
Having at least secondary education	59 (34.5)	75	*χ* ^2^ = 5.22 (0.020)	1.67 (0.095)	
Multigravidity	139 (81.3)	192	*χ* ^2^ = 6.11 (0.013)	2.18 (0.029)	2.41 (1.09, 5.29)

(C) Ready for complications	(*n* = 156) Frequency (%)	Total	Statistical test (*p* value)	*Z* test (*p* value)	OR (95% C.I.)

Age >24 years	94 (60.3)	138	*χ* ^2^ = 4.29 (0.038)	1.49 (0.137)	
Multigravidity	133 (85.3)	192	*χ* ^2^ = 16.65 (0.0001)	4.03 (0.0001)	17.58 (4.36, 70.85)
Hausa/Fulani ethnicity	129 (82.7)	219	Fisher's test (0.001)	1.49 (0.137)	
Having at least secondary education	55 (35.3)	75	*χ* ^2^ = 5.46 (0.020)	2.60 (0.009)	5.15 (1.50, 17.69)
Having good perception of eclampsia	117 (75.0)	167	*χ* ^2^ = 12.58 (0.0001)	0.52 (0.606)	
Being very prepared for birth	144 (92.3)	171	*χ* ^2^ = 109.7 (0.0001)	5.18 (0.0001)	314.87 (35.78, 2771.24)
